# Association Between Fentanyl Use and Post-Intubation Mean Arterial Pressure During Rapid Sequence Intubation: Prospective Observational Study

**DOI:** 10.5811/westjem.18435

**Published:** 2024-11-07

**Authors:** Abdullah Bakhsh, Ahmad Bakhribah, Raghad Alshehri, Nada Alghazzawi, Jehan Alsubhi, Ebtesam Redwan, Yasmin Nour, Ahmed Nashar, Elmoiz Babekir, Mohamed Azzam

**Affiliations:** * Department of Emergency Medicine, Faculty of Medicine, King Abdulaziz University, Jeddah, Saudi Arabia; † Faculty of Medicine, King Abdulaziz University, Jeddah, Saudi Arabia; ‡ Department of Emergency Medicine and Anesthesia Critical Care, College of Medical Sciences, Ibn Sina National College, Jeddah, Saudi Arabia; § Department of Emergency Medicine and Critical Care, Al Habib Medical Group, Jeddah, Saudi Arabia

## Abstract

**Introduction:**

The choice of medications used in rapid sequence intubation (RSI) can result in the difference between an acceptable outcome and a lethal one. When executed properly, RSI is a lifesaving intervention. Nonetheless, RSI may result in fatal complications such as peri-intubation cardiac arrest. The risk of peri-intubation cardiac arrest reportedly increases in patients who are profoundly hypoxic or hypotensive prior to endotracheal intubation. Medication choice for RSI may either optimize or deoptimize hemodynamic parameters, thereby impacting patient outcomes. Therefore, our study aimed to examine the association of change in mean arterial pressure (MAP) with and without the use of a predetermined dose of 50 micrograms (μg) intravenous fentanyl as a pretreatment agent during RSI.

**Methods:**

This prospective observational study included patients undergoing RSI at an academic emergency department (ED) over a three-year period between January 1, 2018–January 1, 2021. Average hemodynamic parameters were measured at the time of induction (prior to medication administration) and 10 minutes after induction. We categorized patients into fentanyl and non-fentanyl groups for analysis, and we compared data using chi-square and *t*-test as appropriate. Logistic regression analysis was conducted to account for potential confounding factors.

**Results:**

A total of 278 patients were included in the analysis, of whom 160 received fentanyl and 118 did not. The majority of the patients underwent RSI by trainees 95.0% of the time. The first-pass success rate was 77.7% in our sample and did not differ significantly between the two groups (*P* = 0.84). Unadjusted analysis showed a larger decrease in hemodynamic parameters in the fentanyl group compared to the non-fentanyl group; systolic blood pressure decreased by 11.2% vs 1.6%, diastolic blood pressure decreased by 13.7% vs 3.8%, and MAP decreased by 12.7% vs 3.2%. After adjusting for potential confounders, fentanyl was 2.14 times more likely to lower MAP by 10%.

**Conclusion:**

The use of 50 μg fentanyl for rapid sequence intubation in an ED is associated with higher odds of decreasing mean arterial pressure by at least 10% at 10 minutes from the time of induction. Therefore, it should be carefully dosed, and its use in clinical practice should be justified to avoid unnecessary complications.

Population Health Research Capsule What do we already know about this issue?

*Peri-intubation hypotension with the use of fentanyl has previously been seen in trauma patients and in the anesthesiology literature.*

What was the research question?

*Was the use of fentanyl as a pretreatment agent during rapid sequence intubation in the ED associated with a change in mean arterial pressure (MAP)?*

What was the major finding of the study?

*Fentanyl was associated with reduced MAP (−12.7% vs −3.2%; P < 0.01), compared to those without fentanyl.*

How does this improve population health?

*Emergency physicians should be aware of the complications associated with fentanyl use in the ED. Careful dosing and clinical justification is advised.*

## INTRODUCTION

Rapid sequence intubation (RSI) is the cornerstone of airway management in the emergency department (ED).^1^ The process involves the administration of an induction agent and a neuromuscular blocking agent to facilitate endotracheal intubation. The primary aim of RSI is to provide optimal tracheal intubation conditions and reduce gastric regurgitation.^2^ Critically ill patients presenting to an ED generally have profound physiological derangements, which are often paired with a rapid decline. The choice of pharmacological agents may optimize or exacerbate the underlying physiology. Therefore, the ideal technique should provide rapid optimal intubation conditions, allowing a high rate of first-pass intubation success while reliably attenuating excessive hemodynamic changes.^3^

When executed properly, RSI is a lifesaving intervention. However, it has been associated with a peri-intubation cardiac arrest rate of 0.9–2.7%.^4^ ^,^ ^5^ In particular, patients with pre-intubation oxygen saturation <90% or pre-intubation systolic blood pressure (SBP) <100 millimeters of mercury (mm Hg) have been reported to have a higher likelihood of peri-intubation cardiac arrest.^5^ Factors related to patient characteristics and procedure technique have a remarkable impact on the rate of occurrence of adverse events. Astute clinicians optimize patient physiology before undertaking RSI to limit complications.

Medications used for RSI have a significant impact on patient outcomes. Fentanyl, an ultra-short-acting pretreatment agent, is used to blunt the catecholamine surge due to α-receptor stimulation from endotracheal intubation. The typical dose is 3–5 micrograms per kilogram (μg/kg) administered intravenously (IV) three minutes prior to intubation.^6^ It is traditionally advocated for patients in whom hypertension can be dangerous, such as those with intracranial hemorrhage, elevated intracranial pressure, ischemic heart disease, and aortic aneurysm/dissection.^6^ We could not find studies reporting the use of fentanyl as pretreatment during RSI by emergency physicians for non-trauma victims. The use of fentanyl, however, is reported in the anesthesiology literature and limited to trauma victims.^7^ ^,^ ^8^ Nonetheless, previous studies have reported that the use of fentanyl as a pretreatment agent is associated with an increased risk of adverse events, such as post-intubation hypotension.^9^ ^,^ ^10^ In fact, post-intubation hypotension is a known risk factor for higher in-hospital mortality rates and prolonged duration of intensive care unit stays.^11^ ^,^ ^12^ ^,^ ^13^

A multicenter, randomized controlled trial compared the use of fentanyl vs placebo with ketamine and rocuronium in patients undergoing RSI in an ED.^14^ The trial’s secondary outcome revealed that 29% of patients in the fentanyl group had at least one SBP measurement <100 mm Hg compared to 16% in the placebo group.^14^ In another study researchers conducted a secondary analysis of data from a multicenter, prospective study of 14 Japanese EDs. They found that patients who received fentanyl had a higher risk of post-intubation hypotension in comparison to those who did not receive the drug.^15^ Previous studies investigated the use of fentanyl with a defined cutoff SBP value. In the present study, we sought to examine the use of 50 μg of IV fentanyl as a pretreatment agent before RSI and its association with percent change in post-intubation mean arterial pressure (MAP).

## METHODS

### Study Design

This single-center, prospective observational study included patients undergoing RSI at an academic ED during a three-year period between January 1, 2018–January 1, 2021. The study protocol was approved by the Unit of Biomedical Ethics at the institution (reg. no.: HA-02-J-008) and was conducted in accordance with the tenets of the Declaration of Helsinki. The need for informed consent was waived due to the observational nature of the study.

### Study Setting

We conducted the study at an academic ED with an annual census of approximately 60,000 including adult, pediatric, and pregnant patients. The ED has an accredited four-year emergency medicine residency training program with a total capacity of 32 residents. Upon acceptance into the program, all residents are required to participate in an airway management workshop. Moreover, residents rotate through the anesthesia department with a focus on elective airway management. The majority of intubations were performed by ED residents under the supervision of board-certified emergency physicians with expertise in RSI. All patients requiring RSI were screened for eligibility. The inclusion criteria were as follows: patients aged ≥18 years; those who were administered both induction and neuromuscular blockades; and those who required emergent tracheal intubation at the discretion of the emergency physician. Patients who did not receive neuromuscular blockades, were <18 years, had received vasopressors for hypotension prior to intubation, and those in whom intubation was performed in settings other than the ED were excluded from the study.

All treatment decisions were made at the discretion of the treating physician and were not influenced by the study. Once the decision to perform RSI was made, patients were prepared in accordance with the following departmental protocol:

1. Non-invasive monitoring of heart rate, systolic, diastolic, mean arterial blood pressure, respiratory rate, 3-lead electrocardiogram, and oxygen saturation using CARESCAPE B650 (GE Healthcare, Chicago, IL) monitors.
2. Placement of two large-bore IV catheters at the level of the antecubital fossa or above.
3. Selection of IV fentanyl (fixed dose of 50 μg) as a pretreatment agent was optional at the discretion of the physician.
4. Selection of an IV induction agent (etomidate 0.3 milligrams per kilogram (mg/kg), ketamine 1 mg/kg, propofol 1 mg/kg, or midazolam 0.3 mg/kg) and an IV neuromuscular blocking agent (succinylcholine 1.5 mg/kg or rocuronium 1.2 mg/kg) based on the physician’s preference. (Dosing may be reduced in hypotension based on physician discretion.)
- *All medications were administered at the same time using the following sequence: pretreatment – induction – neuromuscular blockade, when pretreatment was given; and induction – neuromuscular blockade, when pretreatment was not given*.
5. Optimization of the hemodynamic status to achieve a SBP of at least 100 mm Hg.
6. Optimization of pre-induction oxygen saturation to at least 98% (either via non-rebreather mask at 15 liters per minute or positive pressure ventilation using bag valve mask (BVM) when oxygen saturation dropped below 90%).
7. Placement of the patient in a 20–30^°^ upright position during preoxygenation.
8. Preparation of a suction canister with a Yankauer catheter.
9. Preparation of an intubating device based on the physician’s preference (either direct laryngoscopy using a size 3 or 4 Macintosh blade or video laryngoscopy using a hyperangulated laryngoscope). All endotracheal tubes were loaded with a stylet appropriate for the device used. Adjuncts and backup devices were also prepared, including nasopharyngeal airways, oropharyngeal airways, laryngeal mask airways, and gum-elastic bougies.
10. Confirmation of the position of the endotracheal tube using capnometry or continuous capnography.

Upon completion of a successful endotracheal intubation, the treating physician was required to complete an airway procedure note. At least two registered nurses were to be present during the procedure; one performed documentation, while the other prepared medications. Ultimately, the treating physician was required to enter all the procedure details in the patient’s electronic health record. When entering an electronic procedure note, it was mandatory that the physician include all the details or it might have resulted in the inability to continue patient care via electronic medical records system. This led to a data capture rate of 100%.

An attempt was defined as a laryngoscope blade placed into the oropharynx regardless of whether an endotracheal tube was passed. When oxygen saturation measurements dropped below 90%, the operator removed the laryngoscope blade and provided positive pressure ventilation via a BVM until oxygen saturation reached at least 98%. Modifications in subsequent attempts were made at the discretion of the treating clinician.

### Definition of Post-Intubation Hypotension

Previous studies have defined hypotension as an absolute SBP <90 mm Hg or MAP <65 mm Hg.^16^ It is, however, crucial to note that binary definitions are not always applicable in clinical practice.^17^ Classical anesthesia practice suggests maintaining blood pressure within a relative 20% of the preoperative values.^17^ This is based on the theory that patients with hypertension require higher than normal pressure to adequately perfuse organs that are habituated to high pressure.^17^ In fact, a systematic review^18^ examined the definitions of intraoperative hypotension and found that it could be either based on absolute values (ie, MAP <50 mm Hg, <55 mm Hg, <60 mm Hg, <65 mm Hg, <70 mm Hg, <75 mm Hg) or thresholds relative to baseline preoperative values (ie, decrease in MAP by >10% from baseline, decrease in MAP by >15% from baseline, decrease in MAP by >20% from baseline, decrease in MAP by >25% from baseline, decrease in MAP >30% from baseline). Based on the aforementioned findings, we defined post-intubation hypotension as a decrease in MAP by >10% within 10 minutes of induction.^18^

### Measurements

Once the treating physician decided to proceed with RSI, all patients underwent continuous monitoring of vital signs (heart rate, blood pressure, oxygen saturation, respiratory rate) via non-invasive measures. Blood pressure was measured using a cuff placed around the upper arm and cycled every two minutes. An average of three vital sign readings was obtained at the time of induction (immediately prior to the administration of pretreatment or induction) as a pre-induction value, and 10 minutes after the administration of the first medication in the sequence as a post-intubation value. Adverse events were documented if they occurred within 60 minutes after induction. An individual was assigned to measure the hemodynamic parameters and the time using a stopwatch. Pulmonary aspiration was documented based on chest radiograph evidence of aspiration within 14 days of induction.

### Statistical Analysis

We present categorical variables as frequencies and percentages, while continuous variables are presented as median and interquartile ranges. Patients were categorized into two groups: fentanyl and non-fentanyl. We performed comparisons using chi-square for categorical data and *t*-test for continuous data. We measured percent change in hemodynamic parameters (at induction and 10 minutes after induction) using the following formula: [(post-intubation value − pre-induction value)/pre-induction value] × 100.

We compared percent change in hemodynamic parameters between the two categories using the *t*-test. Binary logistic regression analysis was performed to adjust for potential confounding factors. The dependent variable was at least a 10% reduction in MAP (yes/no), while independent variables included age (in years); weight (in kg); indication for intubation (airway protection [yes/no]); respiratory failure [yes/no]); anticipated deterioration [yes/no]); clinical diagnosis (pulmonary diseases [yes/no]; cardiovascular diseases [yes/no]; hepatic diseases [yes/no]; renal diseases [yes/no]; neurological diseases [yes/no]; endocrine disorders [yes/no]; infectious diseases [yes/no]; toxicity [yes/no]); and choice of medication (fentanyl [yes/no]; etomidate [yes/no]; ketamine [yes/no]; propofol [yes/no]; succinylcholine [yes/no]; rocuronium [yes/no]). Independent variables were selected a priori to avoid overfitting the model.

We categorized presenting conditions into the following: pulmonary diseases (including acute respiratory distress syndrome, pulmonary edema, pulmonary embolism, acute asthma exacerbation, chronic obstructive pulmonary disease exacerbation, pneumothorax, pleural effusion, interstitial lung disease, and hemoptysis); cardiovascular diseases (including acute coronary syndrome, aortic syndromes, dysrhythmias, and circulatory shock due to any cause); hepatic diseases (including acute and chronic liver failure and hematemesis due to liver failure); renal diseases (including end-stage renal disease requiring dialysis and obstructive uropathy); neurological diseases (including status epilepticus, hepatic encephalopathy, uremic encephalopathy, hypertensive encephalopathy, acute ischemic stroke, intracranial hemorrhage, neuromuscular weakness, and altered mental status due to any cause); endocrine disorders (including diabetic ketoacidosis, decompensated hypothyroidism, thyroid storm, and adrenal insufficiency); infectious diseases (including meningitis, encephalitis, cerebral abscess, pyelonephritis, pneumonia, spontaneous bacterial peritonitis, and sepsis from any cause); and toxicity (including opioid toxicity, benzodiazepine toxicity, cocaine toxicity, and alcohol intoxication).

The primary endpoint was at least a 10% change in MAP measured 10 minutes from the time of induction. Level of significance was set at *P* < 0.05.

## RESULTS

**Figure 1.**
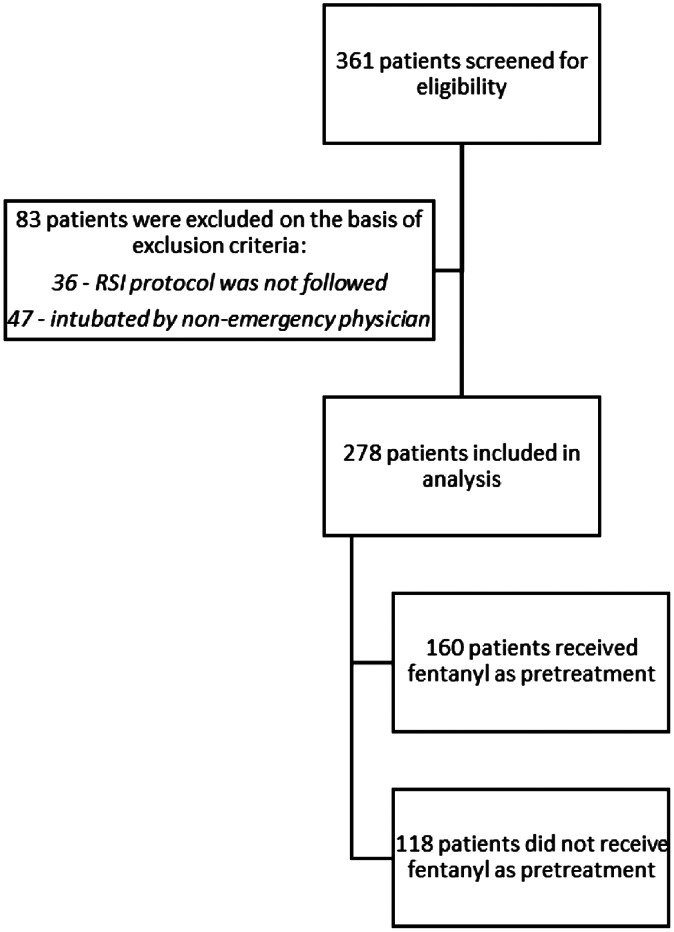
Flow diagram for study participants. *RSI*, rapid sequence intubation.

**Figure 2.**
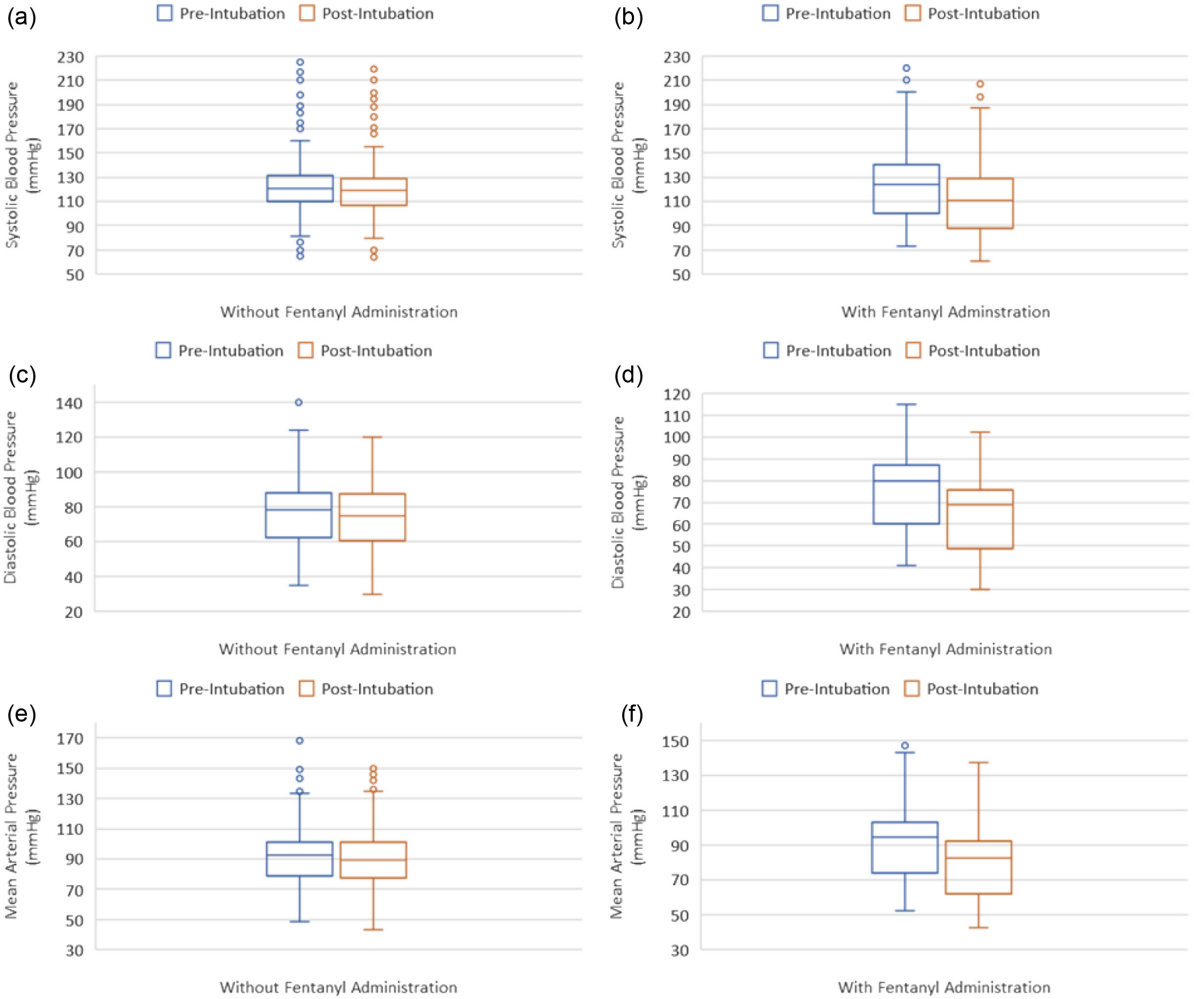
Hemodynamic parameters at the time of induction (pre-intubation) and at 10 minutes after induction (post-intubation) stratified by fentanyl administration: (a) change in systolic blood pressure (SBP) without fentanyl; (b) change in SBP with fentanyl; (c) change in diastolic blood pressure (DBP) without fentanyl; (d) change in DBP with fentanyl; (e) change in mean arterial pressure (MAP) without fentanyl; (f) change in MAP with fentanyl.

During the three-year study period, we collected data from 361 intubation encounters. Of these, only 278 patients met our inclusion criteria (Figure 1). A total of 160 patients received fentanyl as pretreatment, whereas 118 did not receive fentanyl as pretreatment for RSI. Baseline characteristics of the participants (including age, weight, gender, presenting condition, pre-induction vital signs, induction agents, Cormack-Lehane grade, first-pass success rates, device used, and post-induction adverse events) were mostly similar between the two groups (Table 1). However, operators and post-intubation hemodynamic parameters were significantly different between the two groups. A total of 264 (95.0%) patients underwent intubation by trainees. Post-induction median SBP (110 mm Hg [88–128] vs 119 mm Hg [106–129]; *P* = 0.01), diastolic blood pressure (DBP) (69 mmHg [49–75] vs 75 mmHg [60–87]); *P* = < 0.01), and MAP (82 mm Hg [62–95] vs 89 mm Hg [77–100]); *P* < 0.01) were significantly lower in the fentanyl group than in the non-fentanyl group (Table 1 and Figure 2).

**Table 1.** Characteristics of patients undergoing rapid sequence intubation stratified by use of fentanyl.

		All (n = 278)	Pretreatment with fentanyl (n = 160)	No fentanyl (n = 118)	*P*-value
Age (median [IQR])		61 (54–68)	62 (50–71)	60 (56–66)	0.64
Weight (kg) (median [IQR])		78 (73–84)	78.5 (74–85)	77 (71–80)	0.77
Gender	Male	160 (57.6%)	89 (55.6%)	71 (60.2%)	0.44
	Female	118 (42.4%)	71 (44.4%)	47 (39.8%)	
Presenting condition					
Neurologic diseases		85 (30.6%)	53 (33.1%)	32 (27.1%)	0.28
Pulmonary diseases		119 (42.8%)	64 (40%)	55 (46.6%)	0.27
Cardiovascular diseases		38 (13.7%)	24 (15%)	14 (11.9%)	0.45
Hepatic diseases		24 (8.6%)	11 (6.9%)	13 (11%)	0.22
Renal diseases		39 (14.0%)	22 (13.8%)	17 (14.4%)	0.87
Endocrine disorders		25 (9.0%)	14 (8.8%)	11 (9.3%)	0.86
Infectious diseases		71 (25.5%)	39 (24.4%)	32 (27.1%)	0.60
Toxicological causes		3 (1.1%)	1 (0.6%)	2 (1.7%)	0.39
Indication for RSI					
Respiratory failure		120 (43.2%)	64 (40%)	56 (47.5%)	0.21
Airway protection		45 (16.1%)	30 (18.8%)	15 (12.7%)	0.17
Anticipated deterioration		114 (41.0%)	66 (41.3%)	48 (40.7%)	0.92
Pre-induction vital signs					
Pre-induction SBP		122 (106–138)	124 (100–140)	121 (110–131)	0.83
Pre-induction DBP		80 (61–88)	80 (60–87)	78 (62–88)	0.69
Pre-induction MAP		93 (77–102)	94 (73–102)	92 (79–100)	0.75
Pre-induction HR		107 (100–114)	106 (99–112)	107 (100–119)	0.15
Pre-induction RR		24 (22–28)	24 (22–28)	25 (21–29)	0.74
Pre-induction SpO_2_		93 (88–97)	95 (88–97)	91 (88–95)	0.15
Pre-induction GCS		14 (11–15)	13 (10–15)	14 (12–15)	0.09
Post-intubation vital signs					
Post-intubation SBP		115 (96–129)	110 (88–128)	119 (106–129)	0.01
Post-intubation DBP		70 (55–80)	69 (49–75)	75 (60–87)	<0.01
Post-intubation MAP		84 (70–96)	82 (62–92)	89 (77–100)	<0.01
Post-intubation HR		101 (92–116)	93 (88–100)	111 (107–124)	<0.01
Induction agent					
Etomidate		263 (94.6%)	149 (93.1%)	114 (96.6%)	0.20
Ketamine		14 (5.0%)	11 (6.9%)	3 (2.5%)	0.10
Propofol		2 (0.7%)	1 (0.6%)	1 (0.8%)	0.82
Paralytic agent					
Succinylcholine		252 (90.6%)	142 (88.8%)	110 (93.2%)	0.20
Rocuronium		26 (9.4%)	18 (11.3%)	8 (6.8%)	0.20
Cormack-Lehane grade					
Good		235 (84.5%)	137 (85.6%)	98 (83.1%)	0.55
Poor		43 (15.5%)	23 (14.4%)	20 (16.9%)
First pass success		216 (77.7%)	125 (78.1%)	91 (77.1%)	0.84
Device used					
Direct laryngoscopy		29 (10.9%)	19 (11.9%)	10 (8.5%)	0.35
Video laryngoscopy		249 (89.6%)	141 (88.1%)	108 (91.5%)	0.35
Operator					
Physician		14 (5.0%)	12 (7.5%)	2 (1.7%)	0.02
Trainee		264 (95.0%)	148 (92.5%)	116 (98.3%)
Post-induction adverse events					
Cardiac arrest		5 (1.8%)	4 (2.5%)	1 (0.8%)	0.30
Severe hypoxia		14 (5.0%)	10 (6.3%)	4 (3.4%)	0.28
Vasopressor requirement		21 (7.6%)	15 (9.4%)	6 (5.1%)	0.18
Aspiration		5 (1.8%)	2 (1.3%)	3 (2.5%)	0.42
Esophageal intubation		9 (3.2%)	6 (3.8%)	3 (2.5%)	0.57
Right mainstem intubation		7 (2.5%)	3 (1.9%)	4 (3.4%)	0.42
Dental trauma		3 (1.1%)	2 (1.3%)	1 (0.8%)	0.74
Pneumothorax		1 (0.4%)	1 (0.6%)	0 (0%)	0.39
Outcome					
Mortality		20 (7.2%)	12 (7.5%)	8 (6.8%)	0.81

*RSI*, rapid sequence intubation; *IQR*, interquartile range; *SBP*, systolic blood pressure; *DBP*, diastolic blood pressure; *MAP*, mean arterial pressure; *HR*, heart rate; *RR*, respiratory rate; *SpO_2_*, oxygen saturation; *GCS*, Glasgow Coma Scale.

Upon examining percent change in hemodynamic parameters between the two groups (Table 2), we found significantly larger differences in the fentanyl group in SBP (−11.2 vs −1.6%; *P* < 0.01), DBP (−13.7 vs +3.8%; *P* < 0.01), MAP (−12.7% vs −3.2%; *P* < 0.01), and heart rate (12.2% vs +3.7%; *P* < 0.01). [Table 2]

**Table 2.** Change in hemodynamic parameters (between pre-induction and post-intubation values) stratified by use of fentanyl.

	Fentanyl group	Non-fentanyl group	*P*-value
Systolic blood pressure			
Absolute change	−14 mm Hg	−2 mm Hg	*<*0.01
Percent change	11.2%	1.6%	
Diastolic blood pressure			
Absolute change	−11 mm Hg	−3 mm Hg	*<*0.01
Percent change	13.7%	3.8%	
Mean arterial pressure			
Absolute change	−12 mm Hg	−3 mm Hg	*<*0.01
Percent change	12.7%	3.2%	
Heart rate			
Absolute change	−13 BPM	+4 BPM	*<*0.01
Percent change	12.2%	3.7%	

*mm Hg*, millimeters of mercury; *BPM*, beats per minute.

We performed logistic regression analysis (Table 3) to ascertain the association of confounding factors on the likelihood that study participants would sustain at least a 10% reduction in MAP. The model explained 77.1% (Nagelkerke R2) of the variance in post-induction hypotension and correctly classified 88.1% of the cases. Our model showed that the use of fentanyl was 2.14 times (95% confidence interval [CI] 1.34–4.11) more likely to be associated with at least 10% reduction in MAP post-intubation. The remaining predictors did not reveal any association. Based on our post hoc power analysis, the sample size had 86.9% (α = 0.05) power to detect a change in MAP of at least 10%.

**Table 3.** Factors predicting 10% reduction in mean arterial pressure from the time of pretreatment (or induction) until 10 minutes after induction.

	Adjusted odds ratio	95% CI
Age	1.00	*0.98–1.02*
Weight	0.92	*0.73–0.98*
Gender	0.69	*0.40–1.19*
Airway protection	0.40	*0.11–1.41*
Respiratory failure	0.77	*0.33–1.83*
Anticipated deterioration	0.20	*0.11–2.77*
Pulmonary diseases	1.84	*0.83–4.05*
Cardiovascular diseases	1.10	*0.81–3.98*
Hepatic diseases	0.99	*0.39–2.51*
Renal diseases	1.52	*0.71–3.24*
Neurological diseases	1.79	*0.80–3.89*
Endocrine disorders	1.07	*0.42–2.73*
Infectious diseases	1.12	*0.59–2.12*
Toxicological causes	4.36	*0.34–7.70*
Fentanyl	2.14	*1.34–4.11*
Etomidate	1.21	*0.83–1.24*
Ketamine	0.81	*0.76–1.89*
Propofol	1.67	*0.87–2.33*
Midazolam	1.12	*0.91–4.01*
Succinylcholine	0.71	*0.54–2.32*
Rocuronium	0.61	*0.43–1.78*

*CI*, confidence interval.

## DISCUSSION

In this single-center ED study, we found that a decrease in MAP by at least 10% was associated with the use of 50 μg of fentanyl as a pretreatment agent in RSI. This reduction was also seen in SBP and DBP. The reduction in MAP was observed even after adjusting for potential confounding factors. Induction agents have been previously reported to be associated with peri-intubation hypotension.^18^ ^,^ ^19^ Etomidate is least likely to be associated with this outcome.^19^ ^,^ ^20^ ^,^ ^21^ Fentanyl is an optional pretreatment agent; although studies on its use are scant, it is an available option as a pretreatment agent for RSI in some parts of the world and in certain conditions such as trauma.^8^ Studies examining the association between fentanyl use and peri-intubation hypotension are limited.^14^ ^,^ ^15^ While the aforementioned studies defined hypotension based on a cutoff SBP value, in this study we sought to examine the change in hemodynamic parameters from the time of induction until 10 minutes after induction in relation to the use of fentanyl. This approach provides a more dynamic definition of hypotension. Our analysis found at least a 10% reduction in SBP, DBP, and MAP associated with the use of fentanyl.

The purpose of fentanyl is to blunt the sympathetic surge induced by laryngoscopy; thus, dosing should be decided with caution. While lower doses may achieve fentanyl’s purpose, higher doses may introduce unwanted adverse events such as hypotension. We used a standard dose of 50 μg that was administered immediately before the induction agent, and it showed an association with at least 10% reduction in hemodynamic parameters. While in our study we used dosing well below what is described in the literature, a previous study that examined the impact of dose-dependent effects on hemodynamic parameters did not reveal a significant difference between the full and lower dose regimens.^23^ Blood pressure decreased an average of 12 mm Hg (95% CI 7–16) in the full-dose group and by 6 mm Hg (95% CI 1–11) in the reduced-dose group (*P* = 0.10).^22^ Although not significantly different, it is critical to note that hemodynamic parameters showed lower trends in the full-dose group. Therefore, the use of fentanyl should be clinically justified and carefully dosed, especially for critically ill patients who are dependent on their sympathetic drive for survival. Furthermore, its preparation and administration are additional steps in a multistep procedure.

We found a significant difference between operators in terms of the use of fentanyl. While the majority of intubations were performed by trainees, our study showed that attending physicians used fentanyl as a pretreatment agent before RSI more frequently than trainees. This can be attributed to clinical practices developed by attending physicians over their training period. While the physicians staffing our department have been trained in emergency medicine, the majority were trained in the Middle East and some in North American institutions. This may suggest that the use of fentanyl as a pretreatment agent for RSI is variable based on training and practice.

Since the literature does not show a strong benefit in outcomes, (ie, higher first-pass success, reduced mortality rates, reduced esophageal intubation, or reduced hypoxia), we question the use of fentanyl in routine clinical practice. We emphasize the importance of optimizing hemodynamic parameters before RSI and aiming for first-pass success. Therefore, the selection of induction agents that attenuate peri-intubation hypotension during RSI is desirable. A major advantage of etomidate compared with other induction agents is that it preserves cardiovascular stability. It typically does not cause significant hypotension upon induction at a dose of 0.3 μg/kg.^23^ ^,^ ^24^ This is because etomidate does not significantly inhibit sympathetic tone and preserves autonomic reflexes. It is thought that etomidate has this property because it acts as an agonist at the α-2 adrenoreceptors responsible for the peripheral vasoconstriction response to hypotensive effects.^24^ However, there are concerns with its use in critically ill patients because it is known to inhibit adrenal enzymes but is clinically insignificant from a single bolus dose. Other reported side effects include nausea, vomiting, laryngospasm, and myoclonus, all of which can be attenuated by neuromuscular blockade.^23^

Importantly, etomidate did not show increased mortality when compared with other induction agents.^23^ Ketamine may be a reasonable option for RSI because of its quick onset and short duration of action, its preservation of respiratory drive, and its sympathomimetic properties. However, in critically ill patients with depleted catecholamine stores, there is concern for hypotension and cardiac arrest.^23^ Propofol, although having a quick onset and short duration of action, has the most profound effect on blood pressure, which may limit its use in critically ill patients.^23^ Midazolam may be less desirable for RSI as it has a longer onset of action compared with etomidate and ketamine and is a potent venodilator at RSI doses.^23^

Taken as a whole, there was no significant difference between etomidate and other induction agents in the most serious outcome, mortality. In addition, most studies demonstrated favorable peri-intubation hemodynamics with etomidate. Because etomidate is often readily available, clinicians have experience with its use, and its cost is low, it is a reasonable RSI induction agent for critically ill patients.^23^ However, in settings where physicians may not have access to hemodynamically neutral agents such as etomidate for RSI, it would be prudent to optimize patient physiology and hemodynamic parameters during airway management.

## LIMITATIONS

Our study had a few limitations that need consideration. First, this single-center study was conducted in the ED, which limits its generalizability to other settings and institutions. Second, hemodynamic parameters were measured using non-invasive methods, which might have lowered the accuracy of the results. However, this reflects real-world practice due to limited time and resources when performing an arterial line in patients requiring emergent airway intervention. Third, our study lacks valuable hemodynamic data beyond the 10-minute time frame. Fourth, this was an observational study that allowed physician’s discretion. However, a departmental protocol exists for RSI to standardize the procedure.

Fifth, our department protocol allows a maximum dose of 50 μg of IV fentanyl. The dose used in this study is well below the dosing described (3–5 μg/kg) in the literature. Therefore, we were unable to determine the association of hemodynamic parameters with different dosing strategies. Sixth, although data was collected in real time, it was typically entered manually after the procedure, which may have resulted in recall bias. Seventh, the inclusion of induction agent dosing and its association with post-intubation hypotension would add valuable information, but it is not reported in our study due to the variability in dosing between physicians and the extremely low use of certain induction agents such as propofol. Finally, while we performed a logistic regression analysis to account for confounding factors, other unmeasured confounders may still exist given the observational nature of the study.

## CONCLUSION

In this prospective, single-center ED study, we found that the use of 50 μg fentanyl is associated with higher odds of reduction in mean arterial pressure by at least 10%. While fentanyl is an ultra-short-acting agent that could be beneficial for certain conditions, it should be carefully dosed, and its use should be justified in clinical practice to avoid unnecessary complications arising from blood pressure reduction.

## References

[1] Natt B Mosier J . Airway management in the critically ill patient. Curr Anesthesiol Rep. 2021;11(2):116–27. 33897302 10.1007/s40140-021-00448-3 PMC8053895

[2] Cook TM Woodall N Frerk C . Major complications of airway management in the UK: results of the 4^th^ National Audit Project of the Royal College of Anaesthetists and the Difficult Airway Society. Part 1: anaesthesia. Br J Anaesth. 2011;106(5):617–31. 21447488 10.1093/bja/aer058

[3] Lyon RM Perkins ZB Chatterjee D . et al . Significant modification of traditional rapid sequence induction improves safety and effectiveness of pre-hospital trauma anaesthesia. Crit Care. 2015;19(1):134. 25879683 10.1186/s13054-015-0872-2 PMC4391675

[4] Mosier JM Sakles JC Law JA et al . Tracheal intubation in the critically ill. Where we came from and where we should go. Am J Respir Crit Care Med. 2020;201(7):775–88. 31895986 10.1164/rccm.201908-1636CI

[5] April MD Arana A Reynolds JC et al . Peri-intubation cardiac arrest in the emergency department: a National Emergency Airway Registry (NEAR) study. Resuscitation. 2021;162:403–11. 33684505 10.1016/j.resuscitation.2021.02.039

[6] Schofer JM . Premedication during rapid sequence intubation: a necessity or waste of valuable time? Cal J Emerg Med. 2006;7(4):75–9. 20505811 PMC2872531

[7] Wahlen BM El-Menyar A Asim M et al . Rapid sequence induction (RSI) in trauma patients: insights from healthcare providers. World J Emerg Med. 2019;10(1):19–26. 30598714 10.5847/wjem.j.1920-8642.2019.01.003 PMC6264984

[8] Sajayan A Wicker J Ungureanu N et al . Current practice of rapid sequence induction of anaesthesia in the UK: a national survey. Br J Anaesth. 2016;117 Suppl 1:i69–74. 10.1093/bja/aew017 26917599

[9] Griesdale DEG Bosma TL Kurth T . et al . Complications of endotracheal intubation in the critically ill. Intensive Care Med. 2008;34(10):1835–42. 18604519 10.1007/s00134-008-1205-6

[10] Fasting S Gisvold SE . Serious intraoperative problems: a five-year review of 83,844 anesthetics. Can J Anaesth. 2002;49(6):545–53. 12067864 10.1007/BF03017379

[11] Domino KB Posner KL Caplan RA et al . Airway injury during anesthesia: a closed claims analysis. Anesthesiology. 1999;91(6):1703–11. 10598613 10.1097/00000542-199912000-00023

[12] Franklin C Samuel J Hu TC . Life-threatening hypotension associated with emergency intubation and the initiation of mechanical ventilation. Am J Emerg Med. 1994;12(4):425–8. 8031425 10.1016/0735-6757(94)90053-1

[13] Schwartz DE Matthay MA Cohen NH . Death and other complications of emergency airway management in critically ill adults. A prospective investigation of 297 tracheal intubations. Anesthesiology. 1995;82(2):367–76. 7856895 10.1097/00000542-199502000-00007

[14] Ferguson I Buttfield A Burns B et al . Australasian College for Emergency Medicine Clinical Trials Network. Fentanyl versus placebo with ketamine and rocuronium for patients undergoing rapid sequence intubation in the emergency department: the FAKT study-a randomized clinical trial. Acad Emerg Med. 2022;29(6):719–28. 35064992 10.1111/acem.14446 PMC9314707

[15] Takahashi J Goto T Okamoto H et al . Association of fentanyl use in rapid sequence intubation with post-intubation hypotension. Am J Emerg Med. 2018;36(11):2044–49. 29653790 10.1016/j.ajem.2018.03.026

[16] Schenk J van der Ven WH Schuurmans J et al . Definition and incidence of hypotension in intensive care unit patients, an international survey of the European Society of Intensive Care Medicine. J Crit Care. 2021;65:142–8. 34148010 10.1016/j.jcrc.2021.05.023

[17] Salmasi V Maheshwari K Yang D et al . Relationship between intraoperative hypotension, defined by either reduction from baseline or absolute thresholds, and acute kidney and myocardial injury after noncardiac surgery: a retrospective cohort analysis. Anesthesiology. 2017;126(1):47–65. 27792044 10.1097/ALN.0000000000001432

[18] Bijker JB van Klei WA Kappen TH et al . Incidence of intraoperative hypotension as a function of the chosen definition: literature definitions applied to a retrospective cohort using automated data collection. Anesthesiology. 2007;107(2):213–20. 17667564 10.1097/01.anes.0000270724.40897.8e

[19] April MD Arana A Schauer SG et al . Ketamine versus etomidate and peri-intubation hypotension: a National Emergency Airway Registry study. Acad Emerg Med. 2020;27(11):1106–15. 32592205 10.1111/acem.14063

[20] Mohr NM Pape SG Runde D et al . Etomidate use is associated with less hypotension than ketamine for emergency department sepsis intubations: a NEAR cohort study. Acad Emerg Med. 2020;27(11):1140–9. 32602974 10.1111/acem.14070 PMC8711033

[21] Sharda SC Bhatia MS . Etomidate compared to ketamine for induction during rapid sequence intubation: a systematic review and meta-analysis. Indian J Crit Care Med. 2022;26(1):108–13. 35110853 10.5005/jp-journals-10071-24086 PMC8783236

[22] Ter Avest E Ragavan D Griggs J et al . Haemodynamic effects of a prehospital emergency anaesthesia protocol consisting of fentanyl, ketamine and rocuronium in patients with trauma: a retrospective analysis of data from a helicopter emergency medical service. BMJ Open. 2021;11(12):e056487. 10.1136/bmjopen-2021-056487 PMC8689168 34930748

[23] Acquisto N Mosier J Bittner E et al . Society of Critical Care Medicine clinical practice guidelines for rapid sequence intubation in the critically ill adult patient. Crit Care Med. 2023;51(10):1411–30. 37707379 10.1097/CCM.0000000000006000

[24] Valk BI Struys MMRF . Etomidate and its analogs: a review of pharmacokinetics and pharmacodynamics. Clin Pharmacokinet. 2021;60(10):1253–69. 34060021 10.1007/s40262-021-01038-6 PMC8505283

